# Scheme for the Counties: The Needs of London

**Published:** 1919-04-12

**Authors:** 


### AMBULANCE PROVISION.

### Scheme for the Counties: The Needs of London.

The Armistice has been signed, peace is in sight, and probably thousands of Army medical motor-ambulances are lying idle. Such conveyances are badly needed throughout the land for the transit of patients from house to hospital, and it is satisfactory to note that a beginning has been made to apply them to such uses. About 500 Red Cross motor-ambulances have been set apart for a county scheme, which will be under the control of a Home Service Ambulance Committee and worked for a probationary period of twelve months. The County Directors will decide at what towns or villages in their area ambulances are to be stationed. It is suggested that a charge be made for the services of an ambulance. A fee of 1s. 3d. per mile is mentioned, but all would depend upon the necessities of the case and the circumstances of the patient. Ambulances in the country districts, it is hoped, will be placed about thirty miles apart, so that, when needed, one will always be available not more than fifteen miles away, say about an hour's run. Each ambulance is capable of taking four stretcher cases or eight sitters.

### More Ambulances Essential.

For the time being the scheme is to be applied to the counties only; but the great towns have claims also, and no doubt these will receive attention in the not distant future. A relatively small fleet of motor ambulances could be comfortably absorbed by London alone, and would ameliorate the discomforts and sufferings of great numbers of the poor among the population. Although the medical treatment of our great London Hospitals is magnificent, and, from that point of view, leaves little to be desired, the question of suitable transit from house to hospital requires earnest attention and endeavour. We state at once that there is a crying need for more ambulances in hospital medical practice, and we will try to give a few of our reasons.

Let us for a moment review the metamorphosis of transit in the case of street accidents. These were at one time conveyed to hospital on the nearest cart, a hurdle, or the shutter of a shop window. Later the police were made chiefly responsible for these accidents and the sufferer was conveyed to the hospital on a most uncomfortable perambulator, the motive force being the dignified strides of a stolid policeman, and the removal accompanied by a curious crowd of urchins, messenger boys, and the riff-raff of the street. Only after prolonged agitation was the present admirable system of motor transport evolved, by which an accident case can be conveyed to the hospital comfortably, decently, and in the minimum of time.

The attitude of constituted authority towards any radical improvement in medical administration or treatment will appear very puzzling to the "man in the street," but is really quite understandable if inquired into. The root troubles are finance and responsibility, and as such will flourish until we get proper ministerial control of the nation's greatest asset—its health.

The classes of people for whom we claim ambulance conveyance fall into three groups—namely, weakly patients who are to be admitted into hospital when there is a bed vacant for them; weakly people who have to attend hospital as out-patients regularly; and those people actually ill in bed who wish to be conveyed to the hospital for treatment. At present the patients' own legs are often the only means; other are to be acquired only by means of money, a commodity somewhat scarce among the great masses of people suited for hospital treatment. Of course we do not mean to imply that every patient needs conveyance—a healthy man sent with a card of admission for operation on a lipoma of the neck, an inguinal hernia, or suchlike disease. He can quite well walk or go the major part of the journey by tram, electric tube, or motor-omnibus; but in the case of a patient weakened and emaciated by disease, the journey becomes a trying if not an actually dangerous one. In these days travel in London over any distance is associated with a definite amount of mental stress and physical fatigue, greatly augmented in the case of people suffering from heart disease, exopthalmic goitre, and illnesses of kindred gravity, and it is these we must consider.

### Every Hospital should Possess Several.

Then, let us remember that accident which is liable to occur in any household at any time—the abdominal catastrophe. Its possible causes are legion, commonly an acute appendicitis, a strangulated hernia, an intussusception, an acute intestinal obstruction, a ruptured gastric ulcer or ectopic gestation, a twisted ovarian cyst or an obstetric complication which needs immediate hospital treatment. Such catastrophes, as we have said, may occur at any time of the day or night. The general practitioner is powerless to do more than diagnose them, they require immediate operation, for which they need the most rapid possible removal to hospital. Every moment of delay is a moment lost for ever, as the ultimate prognosis depends very much on the speed with which the abdomen is opened and the trouble relieved by the surgeon. At present the overworked practitioner and the harassed family are in great difficulties to find a suitable conveyance. One is probably found at last, but not until precious moments have been lost, even the hiring of a taxi-cab being at present a matter of great difficulty. The modern motor-ambulance is the real panacea to all such difficulties. Every hospital should possess several, and they should be available for use both by night and day.

